# Modelling the influence of temperature and rainfall on the population dynamics of *Anopheles arabiensis*

**DOI:** 10.1186/s12936-016-1411-6

**Published:** 2016-07-15

**Authors:** Gbenga J. Abiodun, Rajendra Maharaj, Peter Witbooi, Kazeem O. Okosun

**Affiliations:** Department of Mathematics and Applied Mathematics, University of the Western Cape, Private Bag X17, Bellville, 7535 Republic of South Africa; Office of Malaria Research, South African Medical Research Council, Durban, Republic of South Africa; Department of Mathematics, Vaal University of Technology, Private Bag X021, Andries Potgieter Blvrd, Vanderbijlpark, 1900 Republic of South Africa

**Keywords:** *Anopheles arabiensis*, Population dynamics, Temperature, Rainfall, Mathematical model

## Abstract

**Background:**

Malaria continues to be one of the most devastating diseases in the world, killing more humans than any other infectious disease. Malaria parasites are entirely dependent on *Anopheles* mosquitoes for transmission. For this reason, vector population dynamics is a crucial determinant of malaria risk. Consequently, it is important to understand the biology of malaria vector mosquitoes in the study of malaria transmission. Temperature and precipitation also play a significant role in both aquatic and adult stages of the *Anopheles*.

**Methods:**

In this study, a climate-based, ordinary-differential-equation model is developed to analyse how temperature and the availability of water affect mosquito population size. In the model, the influence of ambient temperature on the development and the mortality rate of *Anopheles arabiensis* is considered over a region in KwaZulu-Natal Province, South Africa. In particular, the model is used to examine the impact of climatic factors on the gonotrophic cycle and the dynamics of mosquito population over the study region.

**Results:**

The results fairly accurately quantify the seasonality of the population of *An. arabiensis* over the region and also demonstrate the influence of climatic factors on the vector population dynamics. The model simulates the population dynamics of both immature and adult *An. arabiensis*. The simulated larval density produces a curve which is similar to observed data obtained from another study.

**Conclusion:**

The model is efficiently developed to predict *An. arabiensis* population dynamics, and to assess the efficiency of various control strategies. In addition, the model framework is built to accommodate human population dynamics with the ability to predict malaria incidence in future.

**Electronic supplementary material:**

The online version of this article (doi:10.1186/s12936-016-1411-6) contains supplementary material, which is available to authorized users.

## Background

Malaria is still one of the deadliest mosquito-borne diseases in the world. In 2015, an estimated 214 million malaria cases occured, leading to almost 438,000 deaths [1]. Malaria is not present on all continents, mainly occurring in Africa, South–east Asia, Central and South America. It is caused by the protozoan *Plasmodium*, which is transmitted by mosquitoes of the genus *Anopheles* [2–7]. In Africa, three *Anopheles* species, namely *Anopheles gambiae, Anopheles arabiensis* and *Anopheles funestus* are considered to be the major vectors responsible for malaria transmission. The first two species are considered to be the most effective malaria vectors in the world and are classified as a group called *An. gambiae* complex [8, 9]. Also, *An. arabiensis* and *An. funestus* are found in South Africa living in sympatry.

However, malaria as a mosquito-borne disease is strongly influenced by climate variables (temperature, rainfall and humidity). It is well established that weather fluctuations significantly affect not only the life expectancy or completion of the life-cycle of the mosquito, but also the development of sporogonic stages of the malarial parasite within the mosquito’s body [8, 10]. The biting rate and gonotrophic processes are also temperature dependent [7, 8, 11]. For these reasons, a qualitative relationship between the vector abundance and the climate variables can help to identify the peaks of the vector population through meteorological monitoring and forecast [8, 12].

Although, many studies have explored the impact of climate variables on *An. gambiae* at global and regional level, little research has been carried out on *An. arabiensis*. For instance on *An. gambiae*, Ronald Ross [13] developed a simple mathematical model to describe the relationship between the number of mosquitoes and incidence of malaria in humans. Parham and Edwin [14] used published, as well as unpublished field and experimental data to examine the relationships between vector ecology and environmental variables. These relationships are incorporated within a validated deterministic model of *An. gambiae s.s.* population dynamics to offer a valuable tool for highlighting vector response to biotic and abiotic variables. Minakawa et al [15] examined the dynamics of adult *An. gambiae* mosquitoes, their larval habitats, and egg survival potential during the dry season in the basin region of Lake Victoria, western Kenya. In the study, *An. gambiae* showed a strong inclination for wet soil as an oviposition substrate rather than dry soil substrate under the insectary surroundings. Also their findings show that in the dry season, eggs remain latent in the wet soil to resist dryness, and are hatched shortly after they are sufficiently wetted. This suggests why anopheline mosquitoes do not necessarily suffer a severe population bottleneck during the dry season and thus maintain a large effective population size [15]. Craig et al [5] developed a climate-based distribution model to investigate the impact of climate change on *An. gambiae* and malaria transmission over Sub-Saharan Africa. Their model in conjunction with population, morbidity and mortality data is used to estimate the burden of disease and to support strategic control of malaria. Also, Martens et al [16] used a rules-based modelling method to explore how climate change might affect vector abundance and global malaria transmission. Lindsay and Martens [17] extended this study by investigating the implications of climate change scenarios on *An. gambiae* and highland malaria in Africa and, more precisely, in Zimbabwe. Hoshen and Morse [18] also developed a mathematical–biological model, comprising both the climate-dependent within-vector (*An. gambiae s.l.*) stages and the climate-independent within host stages to simulate malaria incidence in Zimbabwe. The model shows a qualitative reconstruction of infection prevalence and a suitable prediction of malaria transmission based on seasonal climate forecasts.

*Anopheles arabiensis* is generally found in Africa, mostly in southern Africa. They live long enough to become infected and infective with *Plasmodium falciparum* [19]. Studies have also shown that their life expectancy is highly influenced by climate variables. In the study of Maharaj [19], it is established in laboratory experiments that *An. arabiensis* feeds and produces eggs but does not oviposit during winter. This is also in line with the study of Omer and Cloudsley-Thompson [20]. Although Le Sueur [21] found some first instar larvae during winter, this suggests that to a lesser extent, oviposition may occur in the field [19, 21]. The laboratory experiments further suggest that *An. arabiensis* could possibly transmit malaria during winter since they do feed during this period. The sporogonic process would be faster during summer than winter period [19]. This suggestion is in line with the previous study of [22] that malaria incidence is directly attributable to the vector feeding habits, abundance and survivorship. However, these studies are laboratory experiments with a limited number of *An. arabiensis* used as samples. Also, the breeding site is assumed to be stagnant. The aim of this study is to develop a deterministic mosquito model that gives a detailed account of the impact of climate variables on the population dynamics of *An. arabiensis*, and to consider a dynamical breeding site being influenced by rainfall and temperature. The laboratory experimental data obtained from the study of Maharaj [19] is used in calibrating the model.

## Methods

### Study area

**Fig. 1:**
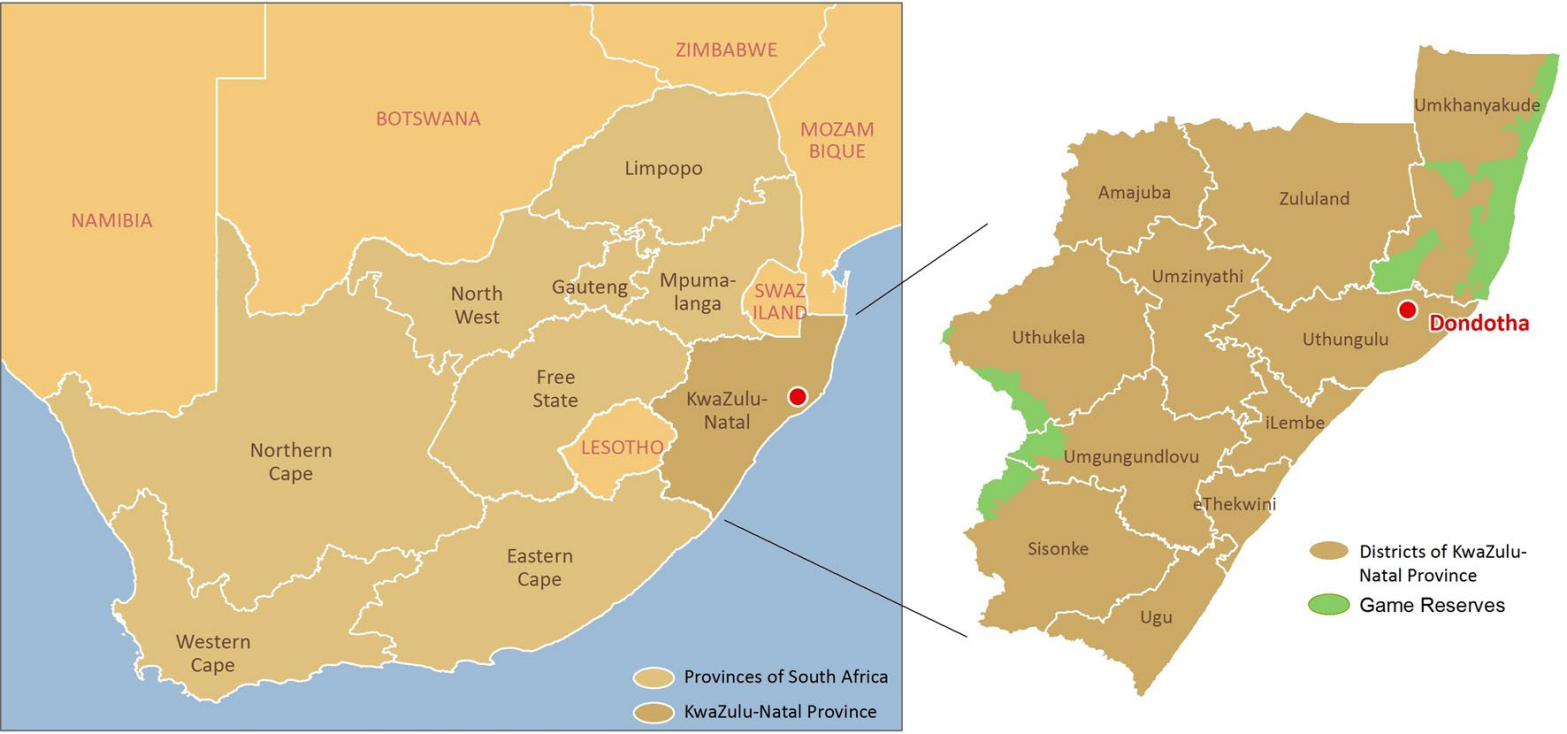
Map showing the location of Dondotha in KwaZulu-Natal Province. *Source*: GIS unit of the Medical Research Council of South Africa

**Fig. 2:**
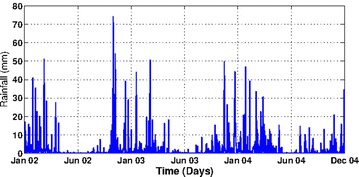
Daily rainfall over calibration period. Showing the daily rainfall of the study area; Dondotha village in KwaZulu Natal Province, South Africa between January 2002 and December 2004

**Fig. 3:**
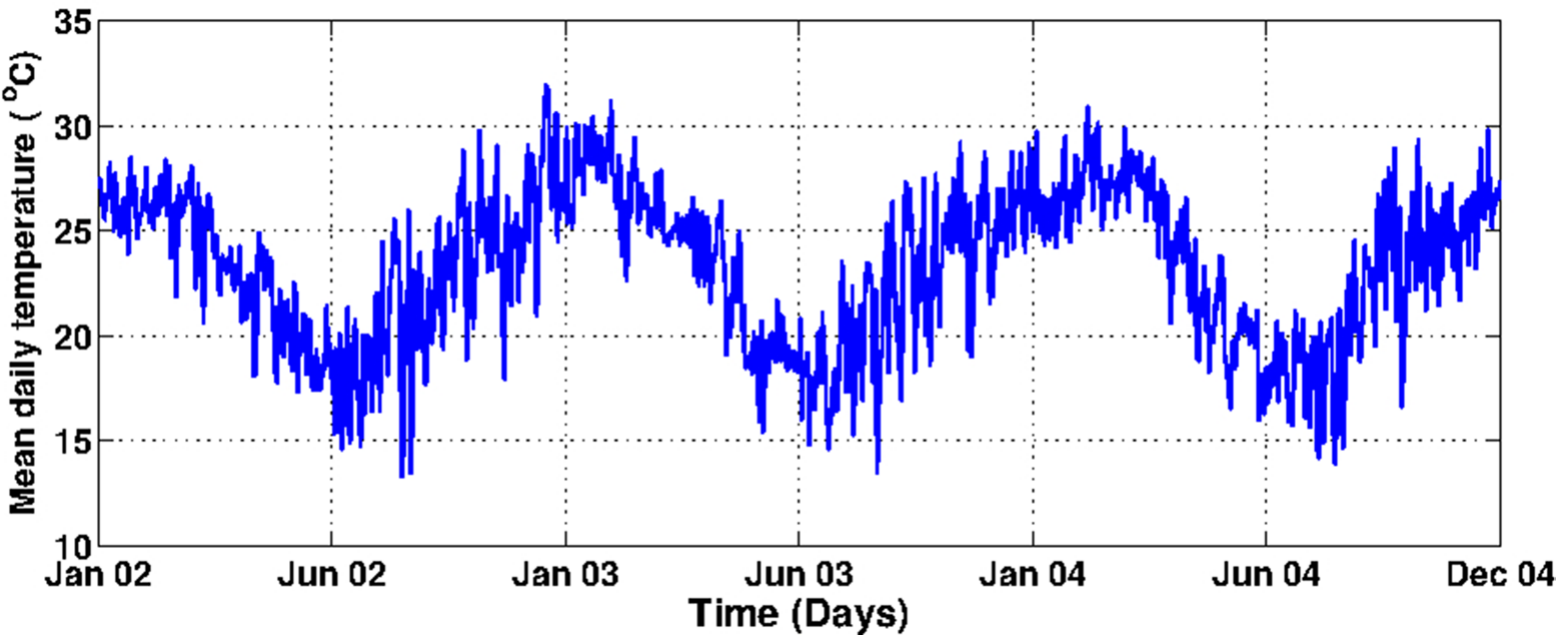
Daily mean temperature over calibration period. Showing the daily mean temperature of the study area; Dondotha village in KwaZulu Natal Province, South Africa between January 2002 and December 2004

The study area is a village called Dondotha in KwaZulu-Natal Province, South Africa. The village (28°34′S, 31°56′E) is situated in the northeast of the province that share borders with three other provinces (Mpumalanga, Free State and Eastern Cape) and countries (Mozambique, Swaziland and Lesotho) as shown in Fig. 1. It experiences long sunny days and dry weather on most days with high rainfall during December–April (see Fig. 2). In the study period (January 2002–December 2004), the heaviest rainfall occurred around December 2002 (78 mm); whereas the highest temperature occurred around January 2003 (mean = 32 °C). Also from Fig. 3, the average daily mean temperature and rainfall increased from January and peaked in February before declining gradually toward June every year.

### Entomological data

The entomological data used in this study is based on laboratory experiments in the study of Maharaj [19]. In the experiment, *An. arabiensis* were collected from the village Dondotha. Fresh breeding stock was caught at the start of each set of experiments and newly laid eggs were kept under insectary conditions of temperature (27 ± 2 °C), relative humidity (70 ± 10 %) and photoperiod (12L:12D with 1 h simulated crepuscular period) [19]. Identification was obtained by using the polymerase chain reaction (PCR) method on samples of the first larval instars of each female [19, 23]. Also, in their studies, all experiments were conducted in a Specht Scientific programmable growth cabinet (model SFPGR066) fitted with a Dumo Dicon P temperature and humidity control unit [19]. The development and survivorship of immature *An. arabiensis* were studied at four fluctuations temperatures. Temperature levels with mean values (17.9, 23.2, 26.1 and 21.4 °C) were used to represent winter, spring, summer and autumn profiles respectively. The output data were used in their study to describe the life table characteristics of *An. arabiensis*.

### Climate data

In the present study, the observational-reanalysis hybrid datasets for the daily precipitation, minimum and maximum daily temperature are considered over the study region for the period 2002–2004. The Princeton University Global Meteorological Forcing Datasets for land surface modelling are produced by the Terrestrial Hydrology Research Group at Princeton University (hereafter, [24]). Also in this study, it is assumed in line with previous studies (e.g., [7, 8]) that the population dynamics of *Anopheles* is mainly driven by two major factors: (1) temperatures—have a strong impact on the survival of *An. arabiensis* populations, and on the development of aquatic stages (e.g., [19]); (2) precipitations—provide breeding sites for immature *Anopheles*. However, excess rainfall can flush away the breeding sites (e.g., [7, 8]).

### Model formulation

**Fig. 4:**
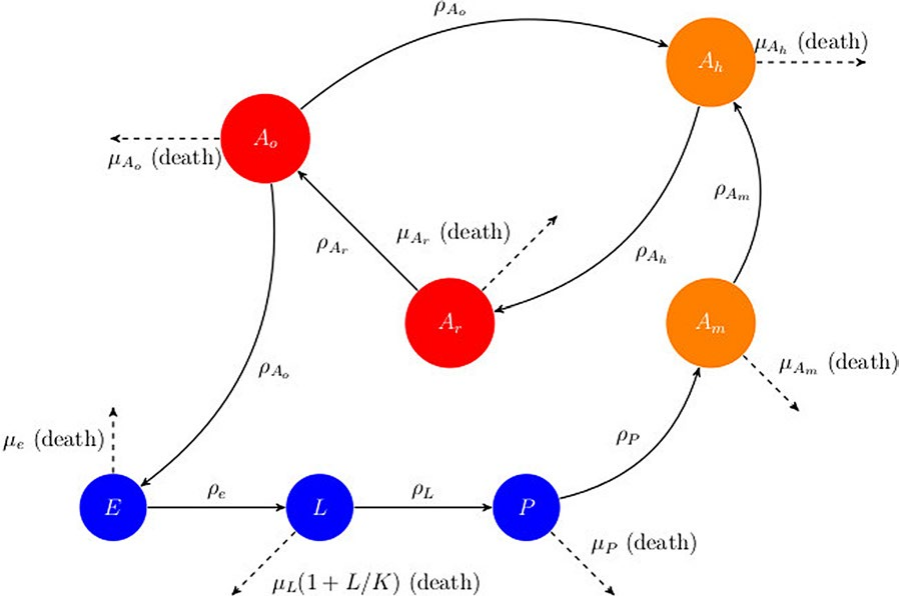
Flow diagram of mosquito population model

The vector population dynamics model used in the present study is based on previously developed models by others [25–28]. The compartmental models of [27] consists of three aquatic stages; eggs (*E*), Larvae (*L*), and Pupae (*P*), and three adult classes; Adult searching for host (*A*_*h*_), Adult at resting state (*A*_*r*_) and Adult searching for oviposition site (*A*_*o*_). One more compartment of adult female *An. arabiensis* searching for mating (*A*_*m*_) is added as shown in Fig. 4. Temperature has a strong impact on the progression rates at the aquatic stage and on the survival of adult populations [29], while rainfall plays a significant role in provision of the breeding sites. In this study, the impact of these factors were incorporated into the model, and additional attention on the dynamics of the mosquito breeding sites (puddle dynamics). Mosquito life begins with eggs, which hatch into larvae under conducive conditions. The larvae further develop into pupae that advance and emerge into adults. Adult female mosquitoes feed on human or animal blood to produce eggs. After biting, the female mosquitoes rest a while in order to develop their eggs. Once the eggs are fully developed, they find a suitable breeding site to lay their eggs and then proceed to find another blood meal. This completes the mosquito feeding cycle [4, 27]. The effects of hibernation and breaks in the reproductive cycle is ignored, and it is assumed that eggs deposited at breeding sites proceed through development immediately (as in [27, 30]. The male population in this model is also overlooked since only female mosquitoes are involved in the transmission of malaria. The seven subgroups have diverse mortality and progression rates. Each subgroup is affected by three processes: (1) increase due to recruitment, (2) decrease due to mortality, and (3) development or progression of survivors into the next state. The parameter *n* is the average number of eggs which are expected to hatch into female mosquitoes laid during an oviposition and $\rho _{A_o}$ (day^−1^) is the rate at which new eggs are oviposited (i.e. reproduction rate). Exit from the egg stage is either due to mortality at $\mu _e$ (day^−1^), or hatching into larvae, $\rho _e$ (day^−1^). In the larval stage, individuals exit by death or progress to pupal stage at a rate, $\rho _L$ (day^−1^). Assuming a stable environment, inter-competition for food and other resources for larvae may occur, leading to density-dependent mortality, $\frac{\mu _LL}{K}$ (day^−1^ mosquito^−1^) or natural death at an intrinsic rate, $\mu _L$ (day^−1^), where *K* is the carrying capacity of the breeding site. Pupae die at a rate, $\mu _P$ (day^−1^), and survivors progress and emerge as adults at a rate $\rho _P$ (day^−1^). In the adult stage, mate seeking mosquitoes die at a rate $\mu _{A_m}$ (day^−1^) while the survivors proceed to search for blood meal at a $\rho _{A_m}$ (day^−1^. Host seeking mosquitoes die at a rate $\mu _{A_h}$ (day^−1^. Those surviving this stage, and if they are successful in feeding, enter the resting stage at a rate $\rho _{A_h}$ (day^−1^. In the resting stage, mosquitoes die at a rate, $\mu _{A_r}$ (day^−1^). Survivors progress to the oviposition site searching stage at a rate $\rho _{A_r}$ (day^−1^). Oviposition site seekers will lay their eggs and return to the host seeking stage or die at a rate $\mu _{A_o}$ (day^−1^). An additional mortality rate of adult mosquitoes $\mu _r$ (day^−1^) related to seeking behaviour is also considered. In line with other studies (e.g., [7, 8]), it is assumed in this study that *Anopheles* female mosquitoes require a blood meal to produce eggs.

Hence, the dynamics of the mosquito population are described by the following system of differential equations:

1 $$\begin{aligned} \begin{array}{ll} \frac{dE}{dt} = n\rho _{A_o}(T_{a}) A_o - (\rho _e(T_{w}) + \mu _e(T_{w}))E \\ \frac{dL}{dt} = \rho _e(T_{w})E - (\rho _L(T_{w}) + \mu _L(T_{w})(1+\frac{L}{K}))L \\ \frac{dP}{dt} = \rho _L(T_{w})L - (\rho _P(T_{w})+ \mu _P(T_{w}))P \\ \frac{dA_m}{dt} = \rho _P(T_{w})P - (\rho _{A_m} + \mu _{A_m}(T_{a}) + \mu _r) A_m \\ \frac{dA_h}{dt} = \rho _{A_m}A_m + \rho _{A_o}(T_{a}) A_o - (\rho _{A_h} + \mu _{A_h}(T_{a}) + \mu _r) A_h \\ \frac{dA_r}{dt} = \rho _{A_h}A_h - (\rho _{A_r} + \mu _{A_r}(T_{a}))A_r \\ \frac{dA_o}{dt} = \rho _{A_r}A_r - (\rho _{A_o}(T_{a}) + \mu _{A_o}(T_{a}) + \mu _r)A_o \end{array} \end{aligned}$$

with initial conditions $E(0), L(0), P(0), A_h(0), A_r(0)$, and $A_o(0)$, where $T_w$ and $T_a$ are water and air temperatures respectively.

#### Puddle dynamics

In this study, it is assumed in line with [31, 32] that the larval carrying capacity *K* is a function of water availability at the breeding site; that is, the water volume of the pond, $V_{pond}$, such that $K = L_{max} \times V_{pond}$, where $L_{max}$ is the maximum larval biomass per surface area. The $L_{max}$ is set to 300 mg m^−2^ following [7, 32]. Although *An. arabiensis* is associated with small ponds [33]. It is also established that, aside rainfall, rivers and human activities (such as irrigation, pipe leakage) could serve as water source to the breeding sites [31, 34]. In an irrigated area, one can expect to have pools of water even outside the raining season. Other studies [7, 16, 35] have also suggested that heavy rainfall can flush off the breeding sites, leading to high larvae mortality. For these reasons, in this study, the puddle dynamics of [7] is considered for the breeding site, that is

2 $$\begin{aligned} \frac{dV_{pond}}{dt}= K_v\left[ R_f (V_{max} - V_{pond})- V_{pond}( \varrho + I_f )\right] , \end{aligned}$$

such that $V_{min} \le V_{pond} \le V_{max}$, where, $V_{min}$ and $V_{max}$ respectively represent the pond minimum and maximum water volume. The rainfall or precipitation rate is denoted as $R_f$, while $K_v$ represents the puddle geometry. Evaporation and infiltration rates are hence denoted by $\varrho$ and $I_f$ respectively. A cylindrical shape puddle of 1.2 m diameter and 0.5 m height is considered for the puddle geometry with the assumption that water depth is much less than puddle height. In line with [7], a fixed constant parameter is assigned for the infiltration rate as shown in Table 1. The evaporation rate by Hamon’s equation [36] is similarly considered as used in other studies (e.g., [37–41]). The effect of waves in the puddle is also ignored since *An. arabiensis* is less common in the areas that are exposed to waves [15].

3 $$\begin{aligned} \varrho = 2.1 \times H_t^2 \times \left( \frac{e_s}{T_a + 273.3}\right) \end{aligned}$$

where, $H_t$ is the average number of daylight hours per day during the month in which day *t* falls. Also, $e_s$ denotes saturation vapor pressure, given by

4 $$\begin{aligned} e_s(T_a) = 0.6108e^{\left( \frac{17.27T_a}{T_a + 237.3} \right) } \end{aligned}$$

In addition to pond dimension the other important parameter of water bodies is the temperature of the water near the surface [7]. Since small ponds and puddles temperature is often one or two degrees warmer than the air temperature [11, 32, 37, 42], it is therefore assumed the temperature of puddles to have a fixed offset relative to the air temperature (such that* T*_*w*_ = *T*_*a*_ + 2 °C).

**Table 1:** Parameters of the model for *An. arabiensis*

Description	Parameters/functional form	Ref.
Number of eggs, n (T_a_)	$-0.61411T_a^3 + 38.93T_a^2 - 801.27T_a + 5391.4$	[19]
Egg development rate, $\rho _e\,(T_w)$	$0.012T_w^3 - 0.81T_w^2 + 18T_w -135.93$	[19]
Larva development rate, $\rho _L\,(T_w)$	$-0.002T_w^3 + 0.14T_w^2 - 3T_w +22$	[19]
Pupa development rate, $\rho _P\,(T_w)$	$-0.0018T_w^3 + 0.12T_w^2 - 2.7T_w +20$	[19]
Egg mortality rate, $\mu _e\,(T_w)$	$0.0033T_w^3 - 0.23T^2 + 5.3T_w -40$	[19]
Larva mortality rate, $\mu _L\,(T_w)$	$0.00081T_w^3 - 0.056T_w^2 + 1.3T_w - 8.6$	[19]
Pupa mortality rate, $\mu _P\,(T_w)$	$0.0034T_w^3 - 0.22T_w^2 - 4.9T_w -34$	[19]
Gonotrophic rate, $\rho _{A_o}\,(T_a)$	$0.00054T_a^3 - 0.038T_a^2 +0.88T_a$	[19]
Adult mortality rate $\mu _{A}\,(T_a)$	$-0.000091T_a^3 + 0.059T_a^2 + 1.3T_a + 9.9$	[2, 16, 48]
Rate adult seeks mating, $\rho _{A_m}$	0.5	Assumed
Rate adult seeks blood meal, $\rho _{A_h}$	0.3–0.5	[27]
Rate adult seeks resting site, $\rho _{A_r}$	0.3–0.5	[27]
Rate adult seeks to mate, $\rho _{A_m}$	0.5	Nominal
Infiltration rate, $I_f$	5 mm / day	[7]
Maximum volume of puddle, $V_{max}$	0.57 m^3^/day	Nominal
Minimum volume of puddle, $V_{min}$	0.001 m^3^/day	Nominal
Daylight hours per day, $H_t$	10–14 hrs/day	Nominal
Maximum larval biomass, $L_{max}$	300 mg m$^{-2}$	[7, 32, 49]

#### Parameters and functions of the model

The parameters used for this model are adopted from the data generated from the laboratory experiments of Maharaj [19]. The extensive data highlight the impact of temperature on developmental attributes of immature *An. arabiensis* under simulated seasonal conditions. The results from the study is used to estimate the parameters and the forcing functions for the gonotrophic rate $(\rho _{A_o})$, development and mortality rate of immature *An. arabiensis*.

**Fig. 5:**
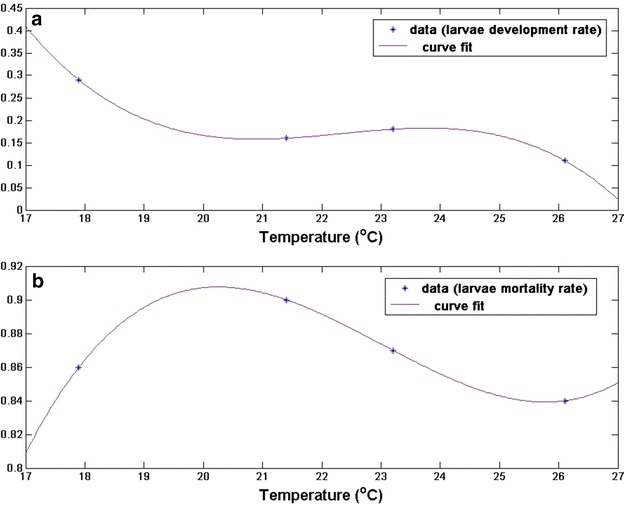
Parameter estimates and curves fit for **a** larvae development rate, **b** larvae mortality rate of *An. arabiensis.* see Additional file 1 for other parameters

Using MATLAB software, the best fitted curves is found (as seen in Fig. 5; Additional file 1) for the gonotrophic rate $(\rho _{A_o})$, development and mortality rate of immature stages. Their parameter functions were further derived as given in the Table 1.

## Results and discussion

### Model validation

**Fig. 6:**
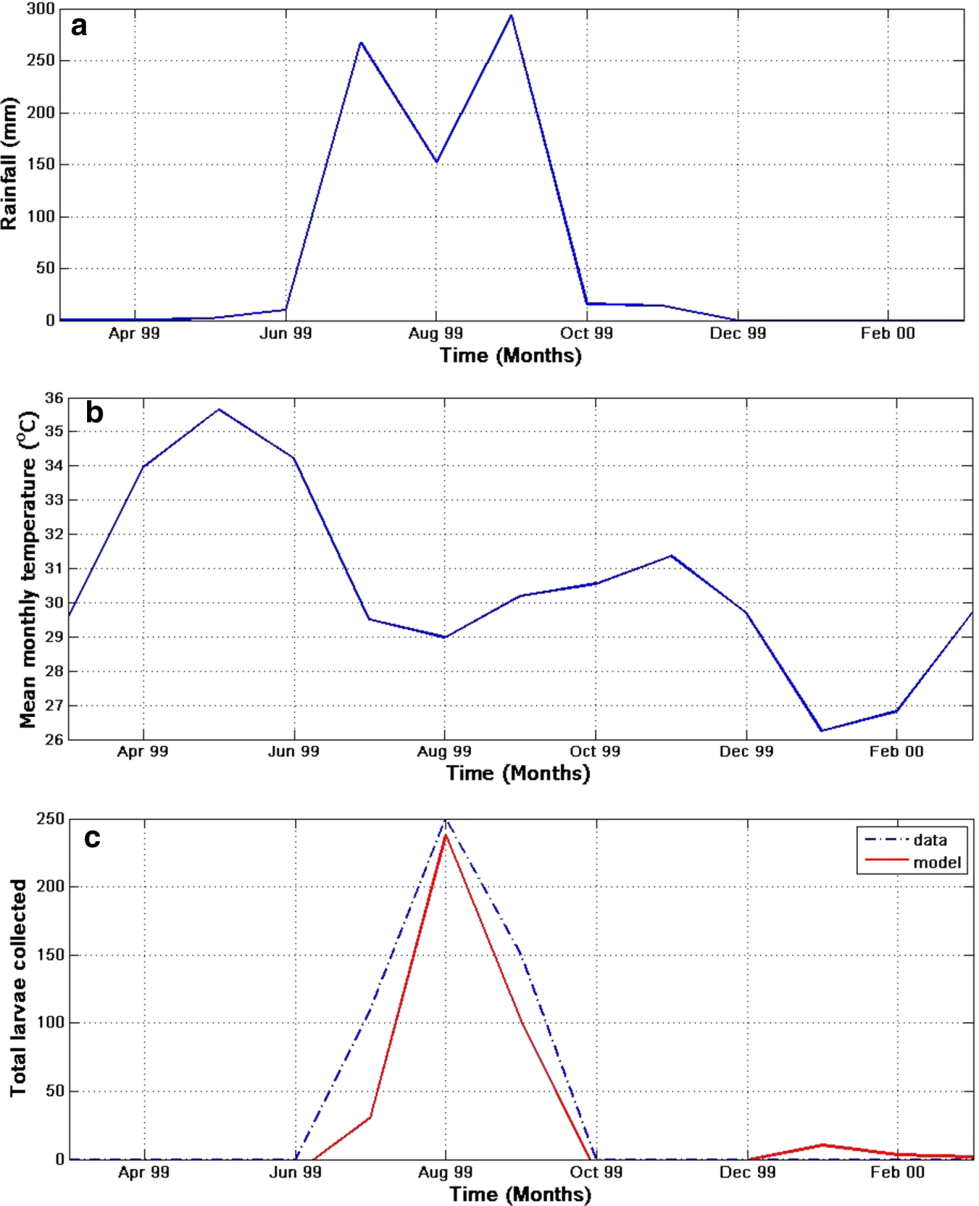
Model validation and climate monthly data of New Halfa town, eastern Sudan. **a** Monthly rainfall, **b** mean monthly temperature, and (**c**) showing the simulated and observed collected larvae over the study area and period

Although it is difficult to find mosquito data to validate the model, in order to ascertain the robustness of the model, the model output is compared with the results obtained from the study of Himeidan and Rayah [43]. In the study, larvae are collected over different breeding sites and sources in New Halfa town, eastern Sudan. The collection was done between March 1999 and March 2000. In the town, temperature is noted to be high in summer (March–June) as shown in Fig. 6b. During this period, rainfall is noted to be minimal (see Fig. 6a). In the raining season (July–September), temperature reaches a minimum as indicated in Fig. 6a. Based on the observed temperature and rainfall during the study period, the dynamics of larvae population at time *t* (red line) is simulated and compared with the mean number of larvae collected (dashed blue line) over New Halfa town as shown in Fig. 6c. The model produce a similar curve (in red) with the observed larvae populations. Also, both graphs (in Fig. 6c) indicate that larvae abundance reaches a minimum between October and June, increases between June and October while reaching the peak in August. The reason for this could easily be linked to low and high rainfall in October–June and June–October respectively. High temperature in summer negatively impacts the larvae and other immature *An. arabiensis* as the breeding sites dry up quickly during this period.

### Sensitivity analysis

#### Model sensitivity to parameters

**Fig. 7:**
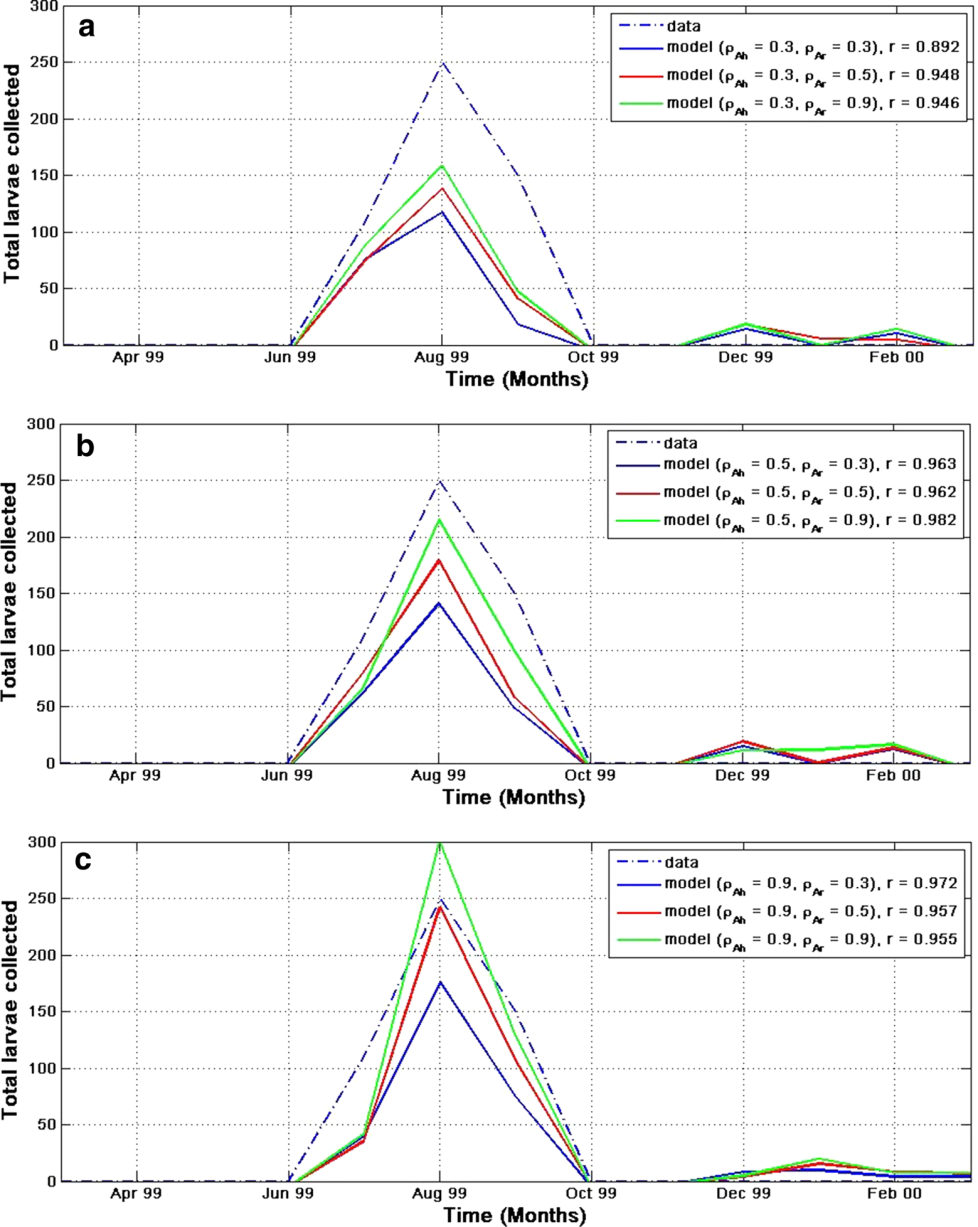
Model sensitivity to parameters. This highlights the sensitivity of the model to parameters when **a** ρ_Ah_ = 0.3, **b** ρ_Ah_ = 0.5, and **c** ρ_Ah_ = 0.9. See main text for details

In this section, the sensitivity of the model is examined with two important parameters, that is, the rate at which host seeking adult *An. arabiensis* enters the resting state $(\rho _{A_h})$ and the rate at which resting adult *An. arabiensis* enters the oviposition searching state $(\rho _{A_r})$. To accomplish this, parameter $\rho _{A_h}$ is held constant at $\rho _{A_h} = 0.3$, while varying $\rho _{A_r}$ between 0.3 and 0.9 in Fig. 7a. Similarly, $\rho _{A_h}$ is held constant at $\rho _{A_h} = 0.5$, as $\rho _{A_r}$ is being varied between 0.3 and 0.9 in Fig. 7b. Finally, in Fig. 7c, $\rho _{A_h}$ is held constant at $\rho _{A_h} = 0.9$ as it varies $\rho _{A_r}$ between 0.3 and 0.9. All figures shows a good correlation between the modelled and observed larvae. Also, the results show that the model is sensitive to both parameters, but more sensitive to $\rho _{A_h}$ than $\rho _{A_r}$. For instance, in Fig. 7a, when $\rho _{A_h} = 0.3$ and $\rho _{A_r} = 0.9$ (in green), there is a significant difference of about 90 larvae between the peaks of the modelled and collected larvae. The peak difference reduces to about 30 larvae when $\rho _{A_h} = 0.5$ and $\rho _{A_r} = 0.9$ in Fig. 7b. The number of simulated larvae overshoots that of observed in Fig. 7c by 50 when $\rho _{A_h} = \rho _{A_h} = 0.9$. For all the simulations, these were considered $\rho _{A_h} = 0.9$ and $\rho _{A_r} = 0.5$ because it produces the closest simulated to observed larvae.

#### Model sensitivity to temperature

**Fig. 8:**
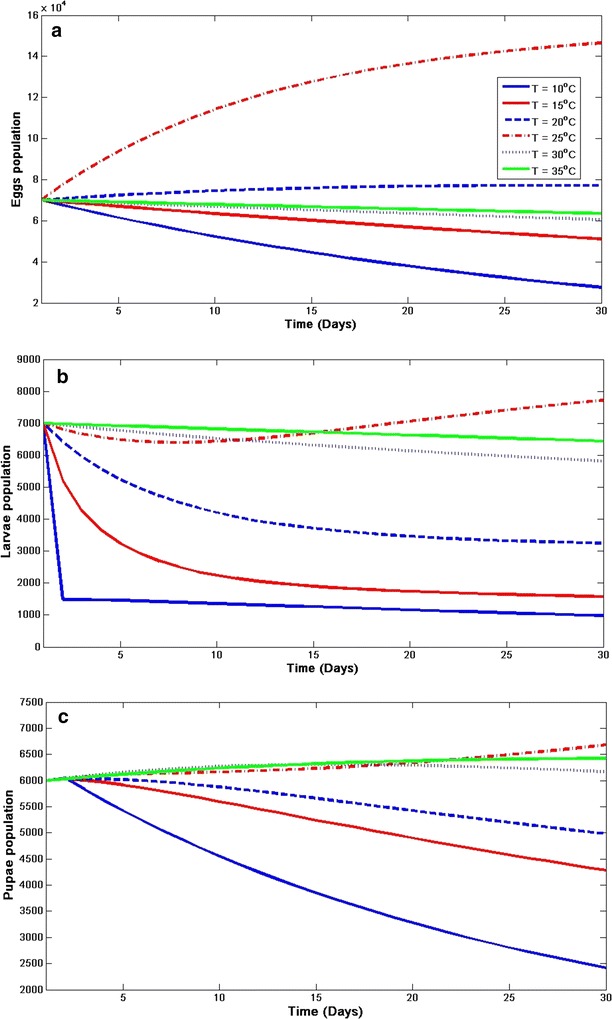
Sensitivity of aquatic-stage mosquito population dynamics to temperature. Effect of constant temperature on **a** eggs, **b** larvae, and **c** pupa of* An. arabiensis*

**Fig. 9:**
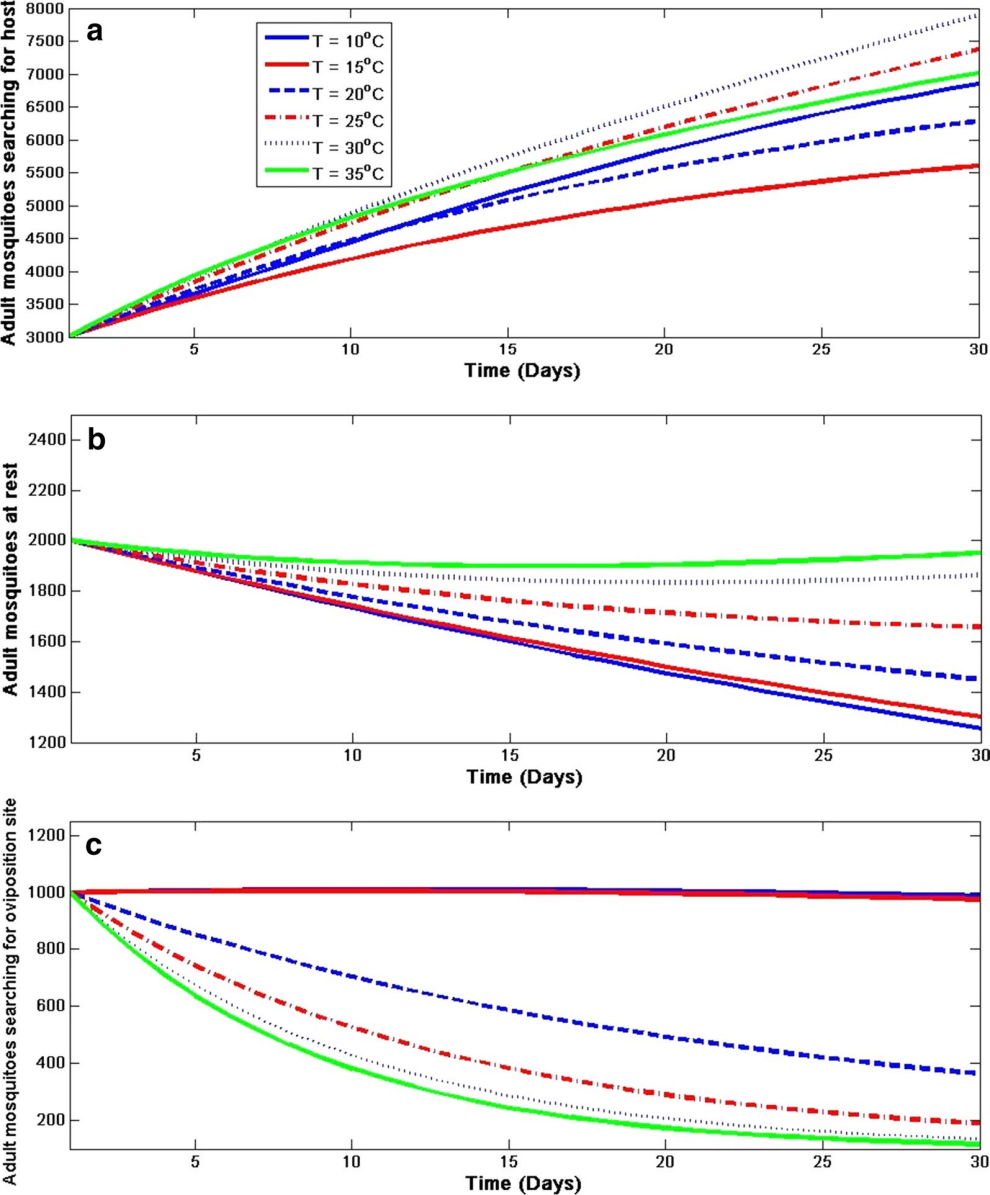
Sensitivity of adult mosquito population dynamics to temperature. Effect of constant temperature on adult *An. arabiensis* **a** searching for host, **b** resting, and **c** searching for oviposition site

For better understanding of the relationship between temperature and vector dynamics, the sensitivity to temperature on both immature and adult mosquito population is examined in Figs. 8 and 9 respectively. To analyse this, it is assumed that the temperature is constant for the first 30 days with varied rainfall. In each class, the dynamics is checked when the temperature is 10, 15, 20, 25C, 30 and 35 °C. It is noticed in both figures (Figs. 8 and 9) that the aquatic mosquitoes are more sensitive to temperature at 25 °C than the adult. It is also noticed that temperature below 15 °C has negative impact on *An. arabiensis*. Consequently, the dynamics are negatively influenced by temperature above 30 °C as specified in other studies (e.g., [7]).

**Fig. 10:**
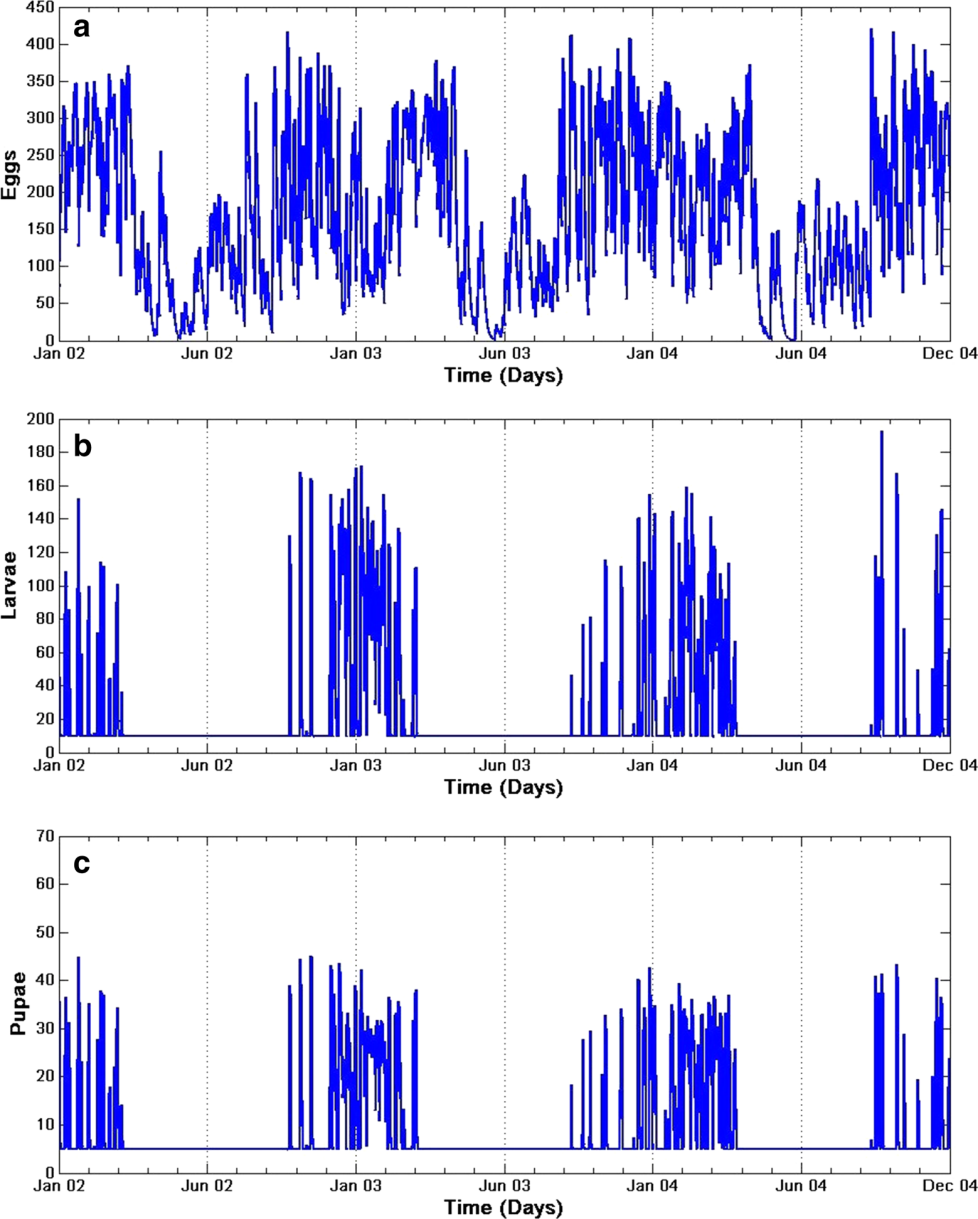
Simulated population of immature *An. arabiensis.* Simulations of **a** eggs, **b** larvae, and **c** pupae population dynamics with climate variables

**Fig. 11:**
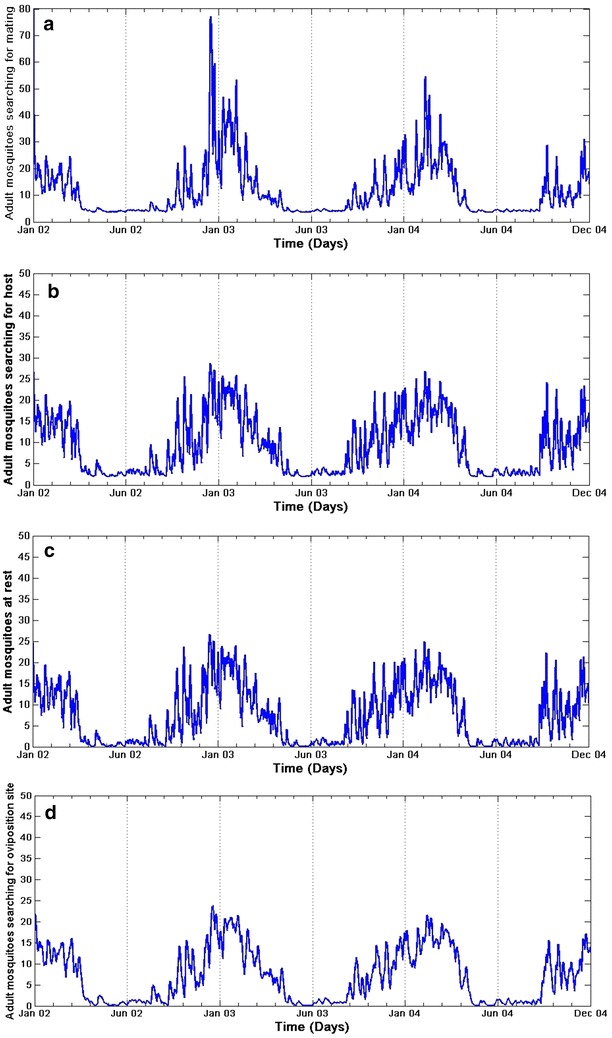
Simulated population of adult *An. arabiensis*. Simulations of adult mosquitoes **a** searching for mating, **b** searching for host, **c** resting, and **d** searching for oviposition site with climate variables

Incorporating the daily climate data of Dondotha between January 2002 and December 2004, the model is used to simulate the dynamics of *An. arabiensis* populations in the region. The model simulates well the abundance of mosquitoes per stage $(E, L, P, A_m, A_h, A_r, A_o)$ over time and presents a strong seasonal variability as shown in Figs. 10 and 11.

With the assumption that the first eggs of the year are laid at the beginning of January, eggs density reaches a maximum in mid-January, February and early March as shown in Fig. 10a. Oviposition activity decreases in between June and mid-August of every year. Larvae and pupae populations follow the same pattern for each year through the study period. Due to human activities such as irrigation and water leakage leading to creation of breeding sites, the model allows the immature *An. arabiensis* population to remain non-zero even in unfavourable conditions between June and August. Differences between years were due to differences in climate variables, the model being otherwise deterministic.

Similar results of the aquatic stages over the adult group were observed as shown in Fig. 11a–d. It is also noted that the adult populations also present a strong seasonal variability with a 6-month period of adult activity as mosquito density is minimal through June, July and August. This suggests that that the number of adults old enough to transmit malaria is intensely influenced by the aquatic stage dynamics, which is in line with the study of [3]. The results also indicate that *An. arabiensis* mosquitoes are present in the region over the study periods, and that the population of *An. arabiensis* in the province is highly seasonal with the peak in summer and minimal in winter as shown in Fig. 11a–d.

Also, temperature is noted to have a stronger influence on adult *An. arabiensis* abundance than precipitation, and it is also the main driver of the model. In fact, most of the mortality and progression rates are temperature-dependent functions. Temperature drives the mortality and transition rates functions in two different ways: higher temperatures favour higher transition rates between stages, although mortality rates decrease with temperature. Yet, according to the simulations in the province, the impact of temperatures is rather favourable to *An. arabiensis* populations as the peak of abundance occurs with the highest temperatures observed in summer period.

**Fig. 12:**
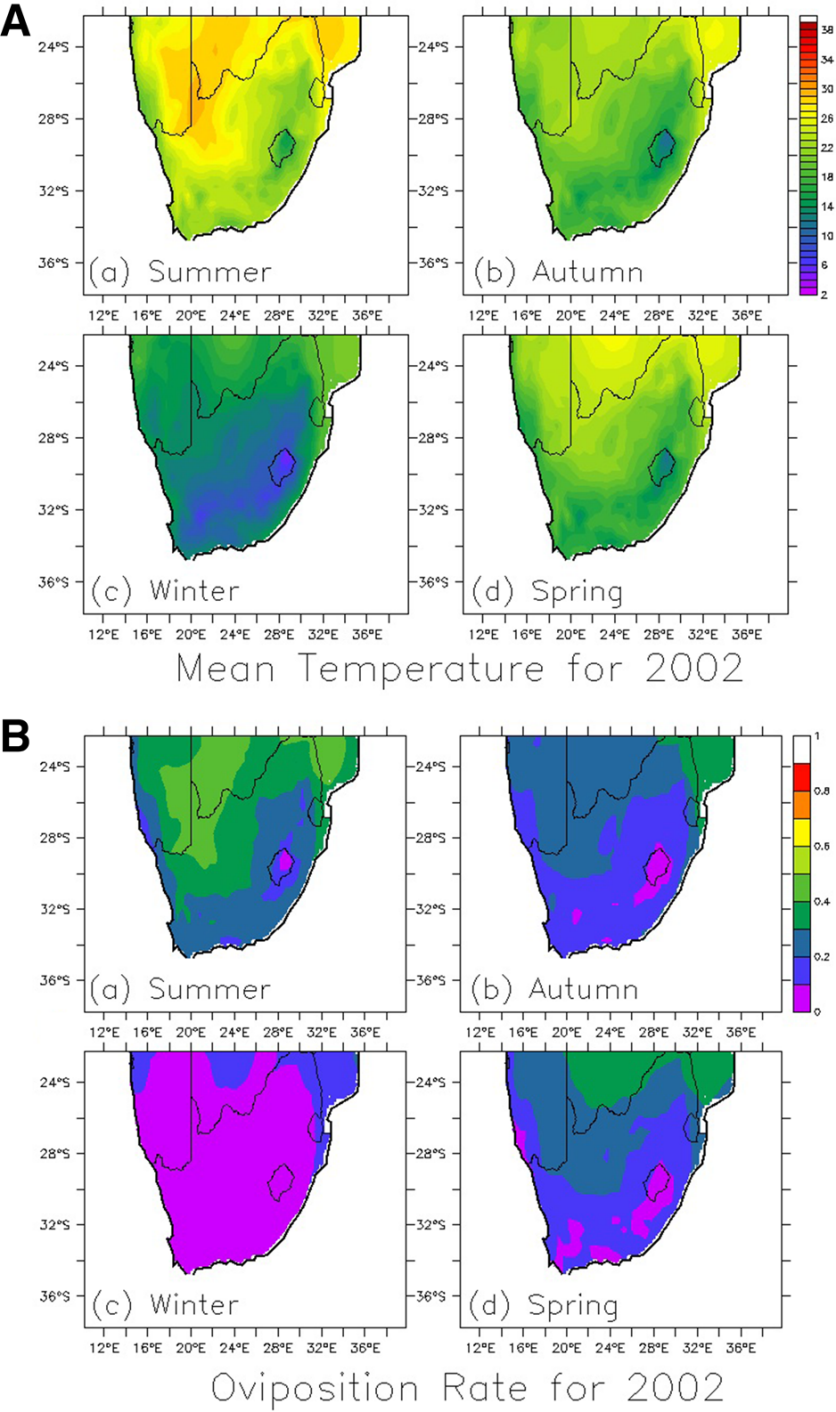
Spatial distribution of temperature and oviposition rate over South Africa. This highlights the spatial distribution of **A** observed temperature, and **B** simulated oviposition rate over South Africa

Running the model over the daily temperature of the 1.0° spatial resolution dataset, the oviposition rate is spatially simulated over South Africa for December 2001– 2002. The results as shown in Fig. 12 suggest why malaria transmission in South Africa is distinctly seasonal. It is noticed that more eggs are produced in summer (December–February) than winter (June–August) period (see Fig. 12B). Some eggs are also produced in Spring (September–November) and Autumn (March–May). This is in line with previous studies [19–21] that *An. arabiensis* do not oviposit in dry and cold conditions. Similarly, as a result of high temperature in summer, it is established that gonotrophic activities is faster during this period as mosquitoes to bite more aggressively for survival and oviposition (e.g., [7]).

## Conclusion

In this paper, a mathematical mosquito model is presented and analysed, this was motivated by the compartmental model of [27]. Two climatic factors (rainfall and temperature) and puddle dynamics are incorporated into the model to understudy the dynamics of immature *An. arabiensis*.

The forcing functions for gonotrophic cycle, progression and mortality rate of eggs, larvae and pupae are also derived from the laboratory experiment in the study of Maharaj [19]. The efficiency of the model are also verified by comparing the simulated larvae with total average number of larvae collected over a town in eastern Sudan from the study of Himeidan and Rayah [43]. Furthermore, the model sensitivity analysis is carried out to examine the sensitivity of the model to parameters.

In addition, the climate data of Dondotha village in KwaZulu-Natal Province are incorporated into the model to simulate the dynamics of the mosquito population over the region. The results highlight the importance of climate on *An. arabiensis* which is accountable for malaria transmission in Africa. It also increases the understanding of significance of the role of mosquito biology in malaria models. The model structure demonstrates a level of robustness as it can be tested on varied climate conditions and on various other species. In particular, the model can be used to study the effect of climate change and variability on vector population dynamics.

Additionally, the model can be developed further by incorporating other processes such as malaria infection. Also, since all mosquito vectors share the same basic life cycle, the model can be converted to other mosquito-borne disease systems, such as Dengue Fever and West Nile Virus. It can be used efficiently as a tool to predict *An. arabiensis* population dynamics. The framework of the model is also designed to accommodate human population dynamics, with the ability to predict malaria incidence in future.

However, the model neglects other important factors influencing the dynamics of the vector population. For instance, humidity has been identified to play a crucial role in both vector and puddle dynamics [31]. Low levels of relative humidity are known to decrease the lifespan of mosquitoes [44]. It has also been established that land cover affects the duration of larval development through its effect on water temperature [45]. Other missing factors in the model includes irrigation [46], deforestation [47], and so on. Hence, the present study leaves these factors for future consideration.

## Additional file

10.1186/s12936-016-1411-6 Model parameterization. Curves fit for gonotrophic rate, development andmortality rate of immature* An. arabiensis*. This figure shows the curve fits for other temperature-dependent parameter. Others are shown inFig. 5 of the main text.

## Abbreviations

An: Anopheles
PCR: polymerase chain reaction
MATLAB: MATrix LABoratory
