# Supplementary figures and images for: Modelling the influence of temperature and rainfall on the population dynamics of Anopheles arabiensis
Source: Malar J. 2016 Jul 15;15:364. doi: 10.1186/s12936-016-1411-6 (PMC4946230; doi:10.1186/s12936-016-1411-6)

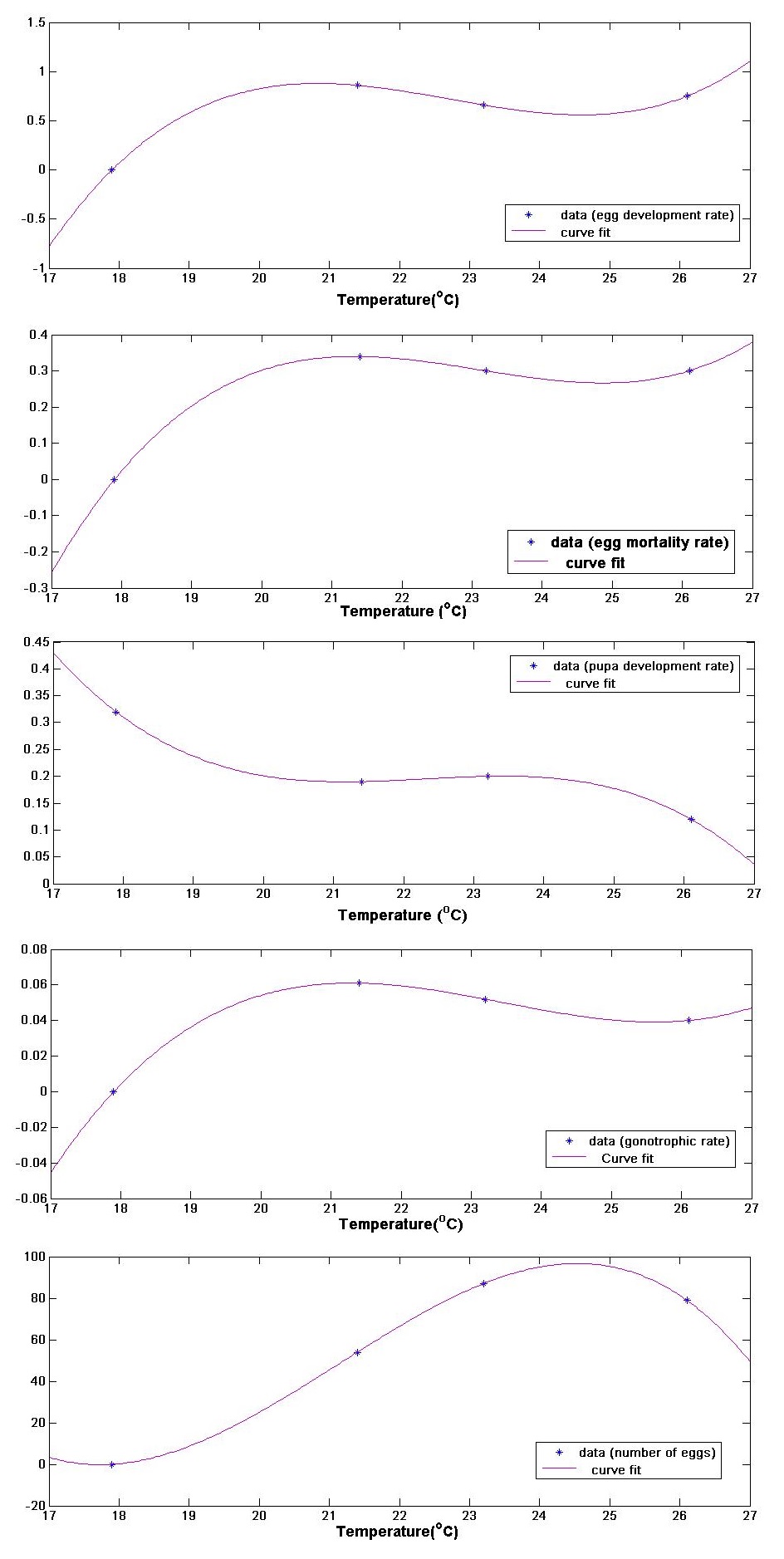

Supplement: Supplementary file 1 — 10.1186/s12936-016-1411-6 Model parameterization. Curves fit for gonotrophic rate, development andmortality rate of immature An. arabiensis. This figure shows the curve fits for other temperature-dependent parameter. Others are shown inFig. 5 of the main text. [file 12936_2016_1411_MOESM1_ESM.jpg]
